# Spotted Hyena skull size variation across geography favors the energetic equivalence rule over Bergmann’s Rule

**DOI:** 10.1093/jmammal/gyae023

**Published:** 2024-04-24

**Authors:** Cybil N Cavalieri, Teresa L McElhinny, Kay E Holekamp, Barbara L Lundrigan

**Affiliations:** Department of Integrative Biology, Michigan State University, East Lansing, MI 48824, United States; Program in Ecology, Evolution, and Behavior, Michigan State University, East Lansing, MI 48824, United States; Michigan State University Museum, Michigan State University, East Lansing, MI 48824, United States; Department of Integrative Biology, Michigan State University, East Lansing, MI 48824, United States; Department of Integrative Biology, Michigan State University, East Lansing, MI 48824, United States; Program in Ecology, Evolution, and Behavior, Michigan State University, East Lansing, MI 48824, United States; Department of Integrative Biology, Michigan State University, East Lansing, MI 48824, United States; Program in Ecology, Evolution, and Behavior, Michigan State University, East Lansing, MI 48824, United States; Michigan State University Museum, Michigan State University, East Lansing, MI 48824, United States

**Keywords:** Bergmann’s Rule, climate, density, ecological rules, energetic equivalence rule, geographic variation, landcover, sexual dimorphism, skull size, Spotted Hyena

## Abstract

Much historic work has focused on establishing geographical and ecological rules that broadly explain patterns in size variation. We examined geographic variation in Spotted Hyena skull size using geometric morphometrics and spatial statistics. We quantified size variation and sexual size dimorphism of the skull, and evaluated the influence of temperature, precipitation, land cover type, and population density on skull size. We found that female spotted hyenas are slightly larger on average than males. Our analysis of regional differences did not indicate geographic variation in sexual size dimorphism. Skull size of Spotted Hyenas varies with geography but does not adhere to Bergmann’s Rule. The smallest individuals of both sexes occur between −5.00° and 10.00° latitude and east of 28.50° longitude, with larger individuals being found elsewhere. Although Spotted Hyena skull size co-varies in some views with such variables as habitat type and climate indicators, skull size in this species most strongly co-varies with population density. The highest population densities are associated with the smallest skull size, possibly reflecting a relationship between high population density and access to resources. These results suggest that geographic variation in Spotted Hyena skull size is better explained by the energetic equivalence rule than Bergmann’s Rule.

## Teaser

New empirical evidence suggests that access to resources influenced by competition and population density, rather than thermoregulatory needs, drives body size in Spotted Hyenas.

## Graphical Abstract

**Graphical Abstract:**
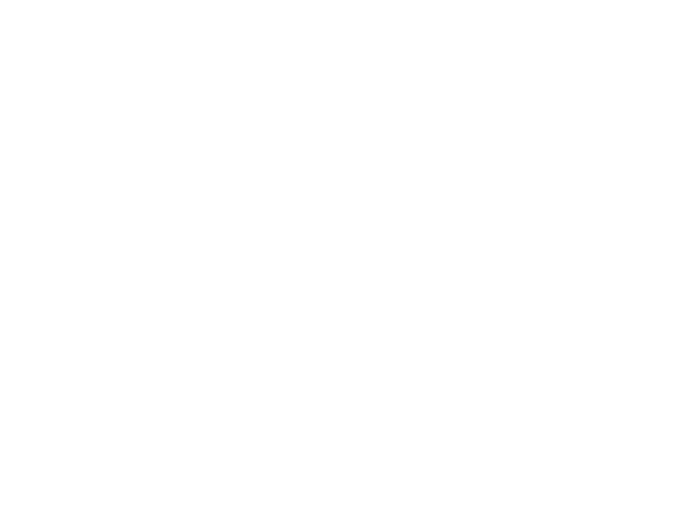

Body size determines strength, complexity, and function of anatomical structures (Bonner 2004; Heim et al. 2017)—while also affecting metabolism and energy balance (Alexander 2005; Speakman 2005; Zhao et al. 2017), and influences the abundance, composition, spatial and temporal distribution of species as well as their interactions (Calder 1996; Brown and West 2000; Smith and Lyons 2013). Unsurprisingly, there is an extensive history of research aimed at identifying factors that influence mammalian body size. Much of this work focuses on establishing geographical and ecological rules that can be applied broadly to explain patterns in size, such as size differences between island and continental species (“The Island Rule”; Foster 1964), the relative size of body extremities in warmer versus colder environments (“Allen’s Rule”; Allen 1877), and variation in size across latitudes (“Bergmann’s Rule”; Bergmann 1847). The oldest of these, Bergmann’s Rule, describes a phenomenon in which endotherms found at higher latitudes or in cooler climates are larger than close relatives from warmer climates (Mayr 1956; Meiri and Thomas 2007; Watt et al. 2010). The premise underlying this rule is that, in cooler climates, larger individuals have a thermoregulatory advantage over those that are smaller because—other things being equal—they have a more favorable surface area-to-volume ratio for retaining heat.

Historically, Bergmann’s Rule has been applied both intraspecifically and interspecifically (Watt et al. 2010) partially due to the prevalence of late 19th-century taxonomists describing species based on small differences, such as slight variation in pelage color, that modern taxonomists would consider within-species geographic variation (Mayr 1956; Laubacher 2011) and partially because Bergmann’s Rule was written in German and not translated into English until 2010 (Watt et al. 2010). In a review of intraspecific body size variation in mammals, Ashton et al. (2000) found broad support for Bergmann’s Rule (78 of 110 species), but not for heat conservation as the primary mechanism. A subsequent compilation of research on geographic size variation in mammalian carnivorans found that only 22 of 44 species (i.e., 50%) conformed to Bergmann’s Rule (Meiri et al. 2004). Body size is under multiple selection pressures simultaneously and the relative strength of those pressures may change multiple times throughout the evolutionary history of a species. Depending on which selection pressure has the strongest influence, a species may exhibit the exact opposite geographic size pattern predicted by Bergmann’s Rule or a heterogeneous geographic size pattern (Mori et al. 2019; Juman et al. 2022; Wood and Cousins 2023).

Availability of resources has been proposed as an alternative explanation for intraspecific geographic variation in mammalian body size; mammals become larger or smaller depending on the size, abundance, and availability of resources (Damuth 1981, 1987; McNab 2010). Several authors have attributed this pattern to reduced access to resources per capita at higher densities, such that more smaller individuals can be supported on limited resources (Peters and Wassenberg 1983; Silva et al. 2001; Loeuille and Loreau 2006). Larger bodies require more resources to maintain (Damuth 1981; Silva et al. 2001), and thus we expect population density to affect body size. This inverse relationship between the body size of an animal species and its population density has been dubbed the “energetic equivalence rule” (Damuth 1981, 1987; Nee et al. 1991).

A relationship between food availability, body size, and population density—though long recognized—has been difficult to quantify because food resources are challenging to measure. Only a few studies (e.g., Dobson and Kjelgaard 1985; Jones et al. 1987; Li et al. 2016) directly examine the relationship between body size and food resources. Most studies rely instead on proxies for food availability including large-scale environmental parameters such as temperature, rainfall, and vegetation (Olalla-Tárraga et al. 2006; Yom-Tov and Geffen 2006; Ferger et al. 2014).

There is strong empirical evidence for an inverse relationship between body size and population density within several large-bodied mammal species. This pattern of small body size associated with higher density is well documented in artiodactyls (e.g., Reindeer, Skogland 1983; Moose, Sand et al. 1995; White-tailed Deer, Simard et al. 2008), probably due to interest in body size management for harvest purposes. Clutton-Brock and Harvey (1977) compared 100 species of primates and found that intraspecific population density is negatively related to body weight.

Some carnivorans also show relatively small body sizes at high population densities. Carbone and Gittleman (2002) found that the number of carnivores supported on a given biomass of prey increases with decreasing body size. In brown bears, females exhibit smaller head circumference—a common proxy for mammal body size—at higher densities (Zedrosser et al. 2006), and in red foxes males and females have shorter body lengths at higher densities (Cavallini 1995) and males aweigh less at higher densities (Cavallini 1995).

Here we investigate intraspecific size variation in a wide-ranging, large-bodied carnivore, the Spotted Hyena, *Crocuta crocuta* (Erxleben 1777; Burgin et al. 2020). This species is suitable for examining geographic size variation, as it is well represented historically and contemporarily in museum collections, and its range extends from the tropics to more seasonal latitudes. Spotted Hyenas occur throughout most of sub-Saharan Africa and are found in a diverse array of habitats including savannas, deserts, swamps, woodlands, and forests up to 4,000 m of elevation (Mills 1990; Mills and Hofer 1998); they occur at very low densities or are absent from dense low-elevation rainforest (Mills and Hofer 1998). Spotted Hyenas are highly social, pack-hunting carnivores (Mills and Hofer 1998) that typically hunt the most abundant medium size ungulate present in their environment (Holekamp and Dloniak 2010), and are known for their ability to crack open large bones to access the nutritious marrow within (Tanner et al. 2008). Although ungulates are an important food source for this species, the diet is remarkably flexible; Spotted Hyenas have been documented feeding on everything from caterpillars to elephants (Mills 1990; Salnicki et al. 2001; Holekamp and Dloniak 2010), facilitating local adaptation to heterogeneous habitats.

Previous studies of spotted hyenas have described marked intraspecific variation (Kruuk 1972; Mills 1990). Indeed, although the species is currently considered monotypic (Burgin et al. 2020; Lewis and Werdelin 2022), historically as many as 21 subspecies were described based on slight variation in skeletal morphology and pelage color (Heller 1914; Allen et al. 1924; Meester et al. 1986). In a taxonomic review of Spotted Hyenas, Matthews (1939) assessed morphological variation and determined that the different morphologies observed could not be geographically delineated, precluding the use of subspecies. Several researchers have noted geographic clines in Spotted Hyena size including an equatorial cline characterized by smaller individuals at the equator and larger individuals to the north and south (Kurten 1957; Klein and Scott 1989; Mills 1990), and a south-to-east cline with heavier individuals in the south and lighter individuals in montane eastern Africa (Sillero-Zubiri and Gottelli 1992). Some authors (Kurten 1957; Klein and Scott 1989) have suggested that these clines in body size largely reflect the influence of temperature (an example of Bergmann’s Rule). But another plausible explanation for observed relationships between body size measures and locality is that they reflect geographic variation in resource availability.

Intraspecific differences in body size can also be the result of sexual selection (Ralls 1976; Shine 1988). Spotted Hyenas are especially interesting with respect to sex roles in that they have a fission–fusion social structure with a linear dominance hierarchy in which females and their offspring are dominant to breeding immigrant males (Frank 1986; Smale et al. 1993). Female dominance over males is rare in mammals (Ralls 1976; Gittleman and Valkenburgh 1997; Meiri et al. 2005; Bidau and Martinez 2016). In the most extensive study to date of sexual dimorphism in this species, Swanson et al. (2013) examined linear measurements taken from 651 live animals immobilized in the Masai Mara National Reserve, Kenya, and found that females had slightly but significantly larger cranial dimensions reflecting differences in the musculature and bones associated with bite force (zygomatic arch to the top of the sagittal crest, zygomatic arch to the back of the sagittal crest, and head circumference). Spotted Hyena skulls take a long time to mature, presumably reflecting the need to cope with the durophagous diet ingested by adults, suggesting that female social dominance in this species may reflect selection pressure on mothers to help offspring with an immature feeding apparatus obtain access to food in a highly competitive environment (Watts et al. 2009; Tanner et al. 2010). If so, the degree of skull sexual dimorphism might be expected to co-vary positively with group size or density.

In this study, we investigate geographic variation in Spotted Hyena size using a large sample from across the range of the species. We use skull size as a proxy for body size because body size data are more difficult to obtain and are usually unavailable for Spotted Hyenas collected outside of East Africa. We first quantify the degree of sexual size dimorphism in Spotted Hyenas. Next, we evaluate the influence of temperature, precipitation, land cover types, and population density on skull size using temperature and precipitation indices as surrogates for thermoregulatory demands, and water availability and land cover type as proxies for habitat. Finally, we ask whether geographic variation in Spotted Hyena skull size is explained by resource availability using population density as an indicator reflecting intraspecific competition and access to resources. We make 3 explicit predictions about the expected relationships between Spotted Hyena skull size and our other variables: first, we expect female-biased sexual size dimorphism in Spotted Hyena skull size in accordance with previous investigations; second, we expect Spotted Hyena skull size to increase as temperatures decrease consistent with the principles of Bergmann’s Rule; third, we predict a decrease in Spotted Hyena skull size in response to heightened competition for resources, in accordance with the energetic equivalence rule.

## Materials and methods

### Specimens

**Fig. 1.**
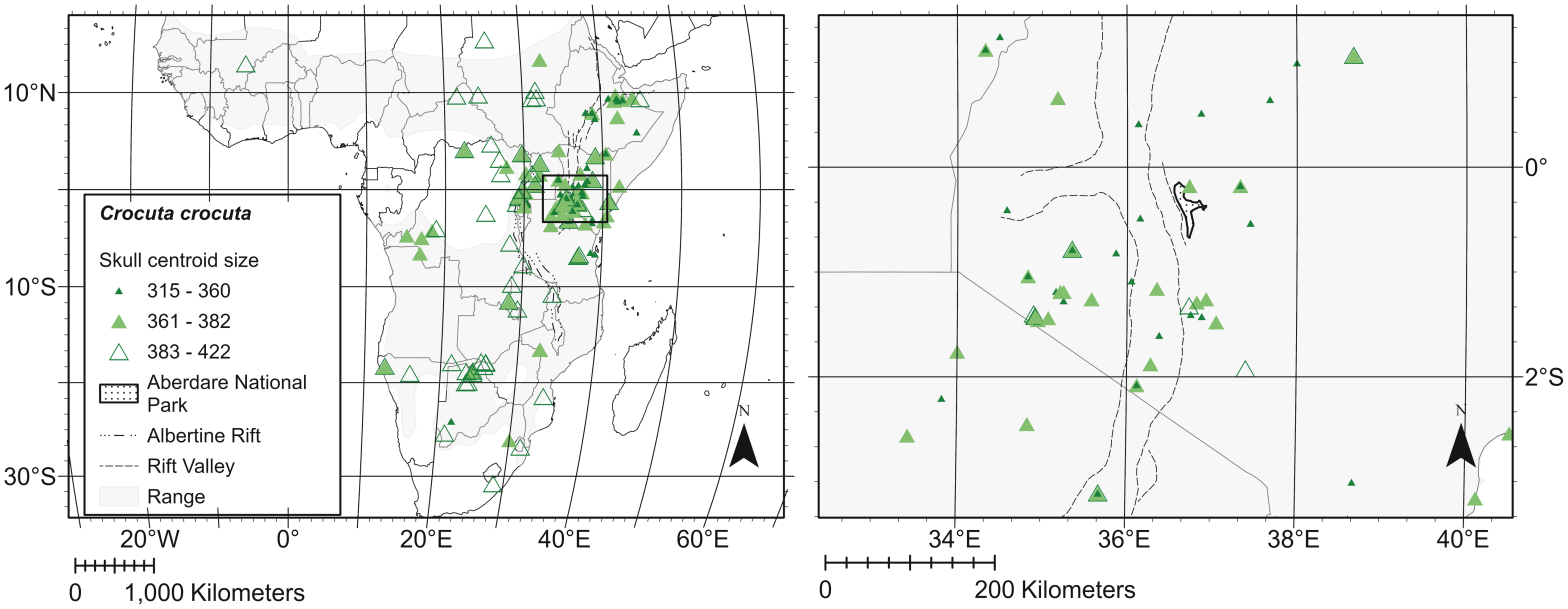
Left panel: Specimen collecting localities (triangles) for *Crocuta crocuta* skulls used in this study. Triangle represents centroid sizes: 315 to 360 dark green closed triangle; 361 to 382 light green closed triangle; 383 to 422 dark green open triangle. The Rift Valley is indicated by a dashed line and the Albertine Rift by a dashed and dotted line. The current geographic range for *C. crocuta* is shaded in gray. The Aberdare National Park is indicated by a stippled area. The right panel is magnified to show detail indicated by the open black square in the left panel.

The sample comprised 332 skulls of adult spotted hyenas (121 females, 125 males, 86 of unknown sex) obtained from 14 natural history collections (Supplementary Data SD4–SD6). Full maturity was defined by complete eruption of permanent teeth, and complete—or nearly complete—closure of the lambdoid and basilar sutures (McElhinny 2009). Specimens were collected from the field between 1900 and 2006 and include individuals from 21 sub-Saharan Africa countries (Fig. 1). The distribution of collecting localities encompasses much of the current geographical range of *C. crocuta*, as well as regions in south and central Africa where *C. crocuta* appears to have been recently extirpated (IUCN 2021). The Sahel, tropical West Africa, Angola, and western Zambia are not as well represented as East Africa (i.e., Tanzania, Kenya, Uganda, Rwanda, and Burundi). Collection data for each specimen, including date of collection, locality (with elevation), and sex (determined at the time of collection), were obtained from museum records. Specimens without coordinate data were georeferenced from specimen locality data using established guidelines from Chapman and Wieczorek (2006) and the georeferencing software GEOLocate Web Application (Rios 2019). For specimens missing elevation data, elevation was extracted from a digital elevation model using ArcGIS Pro 2.9.4 and coordinates obtained from georeferencing. Exploratory models indicated no relationship between elevation and skull size, and thus elevation was eliminated from subsequent analyses, reducing the number of parameters and helping to prevent overparameterization of models (Esri 2020).

### Morphological data

**Fig. 2.**
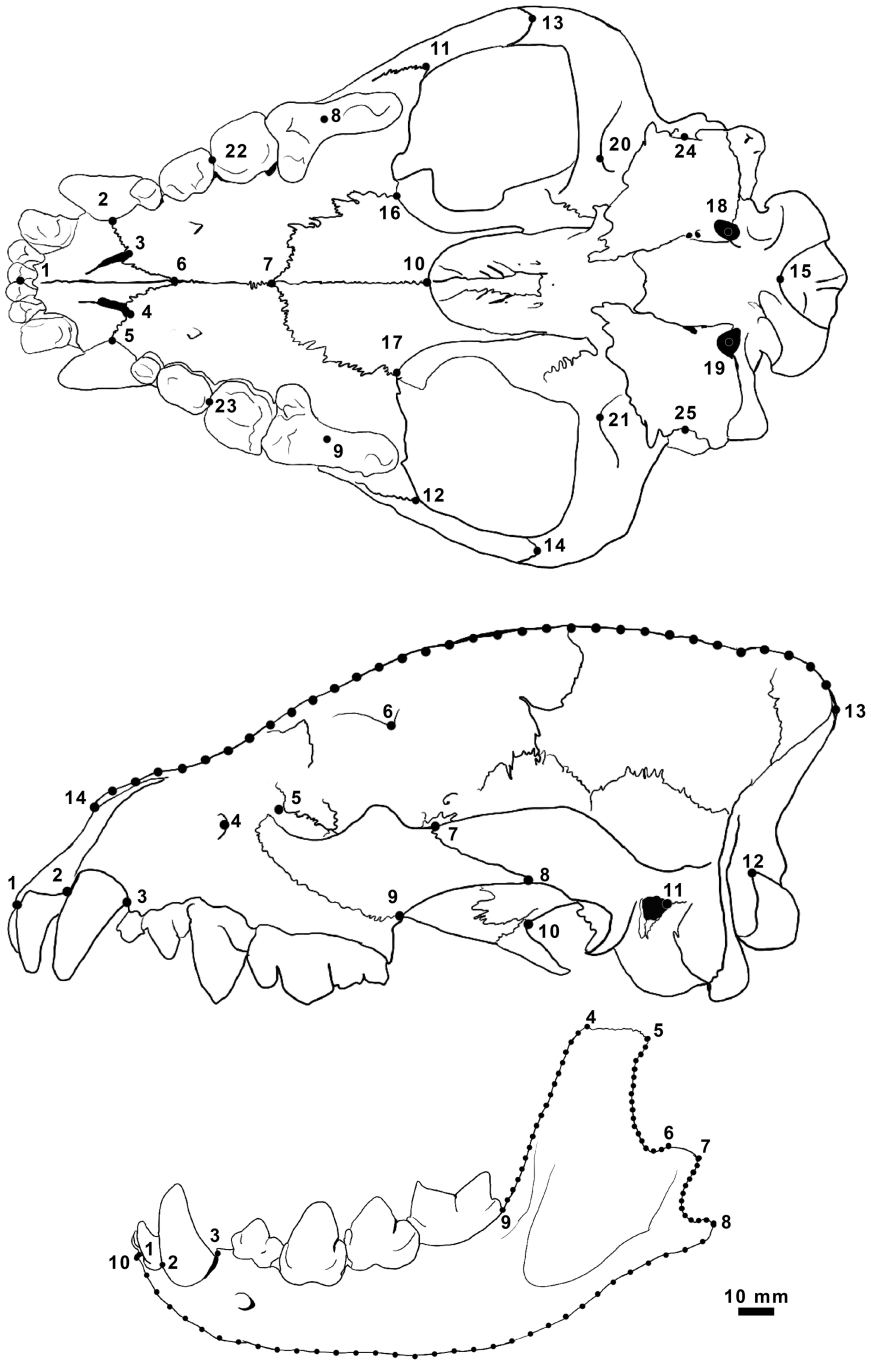
Position of landmarks and semilandmarks on a skull of Spotted Hyena (*Crocuta crocuta*) for the ventral cranium, lateral cranium, and lateral mandible. Landmarks and semilandmarks are represented by black points and landmarks are numbered.

Skulls were photographed using a digital camera in views: ventral cranium, lateral cranium, and lateral mandible (Fig. 2). Images of the cranium were obtained by orienting specimens: in ventral view with the palate parallel to the photographic plane; in lateral view with the midsagittal plane parallel to the photographic plane; and in lateral view of mandible with the long axis of the dentary parallel to the photographic plane. A 10-mm scale was included in all photographs.

To quantify variation in skull size, we used 2D landmark-based geometric morphometrics. Landmarks and semilandmarks (Fig. 2; Supplementary Data SD1–SD3) selected to capture overall size (Zelditch et al. 2012) were digitized by the same observer (CNC) using tpsDig2.32 (Rohlf 2015). For each view, landmark configurations were superimposed to remove variation in scale, position, and orientation by a generalized least-squares Procrustes superimposition using the “geomorph” package (Adams and Otárola-Castillo 2013; Adams et al. 2015) in R version 3.4.1 (R Core Team 2017). Semilandmarks contain an additional nuisance parameter (position along the curve) that was removed by sliding them to minimize bending energy (Green 1996; Bookstein 1997). We used centroid size as the size metric in all analyses. Centroid size is the square root of the summed squared distances of each landmark from the centroid of the landmark configuration; with strategic placement of landmarks, it better captures the overall size of an object than linear measurements (Zelditch et al. 2012).

### Bioclimatic variables

Bioclimatic variables were obtained for the collection locality of each specimen. Annual mean temperature and annual precipitation were extracted at a spatial resolution of 10 min (~340 km^2^) from the WorldClim global climate database (Fick and Hijmans 2017). To capture habitat variation, 28 vegetation types were extracted from the land cover map of Africa at a spatial resolution of 1 km (Mayaux et al. 2004) and collapsed into the 4 broad land cover types forest, mixed grassland and forest, grassland, and bare ground. We acknowledge that there is a temporal mismatch between our skull sample (collection years 1900 to 2013) and the land cover (2000) and climate data (WorldClim 1.4: current conditions ~1960 to 1990) used in this analysis (Mayaux et al. 2004; Fick and Hijmans 2017). Although “historical” environmental data are available for Africa (e.g., HYDE 3.1 and CRU TS version 4), those data are highly interpolated due to the small number of sample sites and variables included are not directly comparable to those used in our analyses—hence, we were not able to incorporate them here. Any resulting temporal mismatches in conditions experienced by the hyenas in our sample might weaken overall morphological patterns, but seem unlikely to produce misleading results.

### Hyena density data

**Fig. 3.**
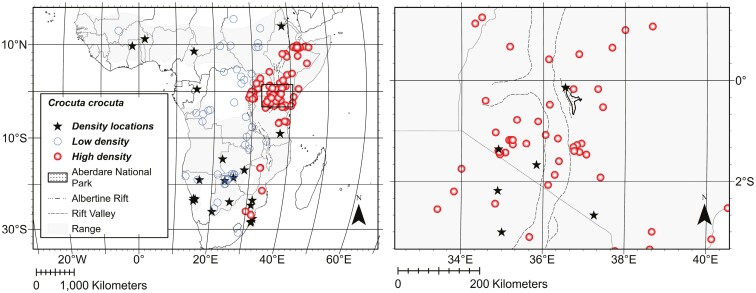
Left panel: Locations for Spotted Hyena (*Crocuta crocuta*) density estimates listed in Table 1 (black stars) and interpolated density estimates corresponding with specimen collecting sites; open blue circles low density (0.0045 to 0.02 Spotted Hyena/km^2^, *n* = 60) and open red circles high density (0.03 to 1.65 Spotted Hyena/km^2^, *n* = 260). The Rift Valley is indicated by a dashed line and the Albertine Rift by a dashed and dotted line. The current geographic range for *C. crocuta* is shaded in gray. The Aberdare National Park is indicated by a stippled area. The right panel is magnified to show detail indicated by the open black square in the left panel.

Spotted Hyena density data (individuals/km^2^; Table 1) were obtained from a compilation by Holekamp and Dloniak (2010) that was updated to include additional information subsequent to 2010. All density data are based on published field studies, PhD dissertations, or MS theses. Density estimates are geographically widespread but correspond only roughly to the regions where our specimens were obtained (Fig. 3). Davis et al. (2022) raised concerns about the accuracy of some methods used to estimate population density in spotted hyenas. In order to evaluate whether density data based on less robust methods (e.g., call-in surveys and track counts) unduly influenced our analysis, we compared the pattern of Spotted Hyena density across Africa based on those data to those based on a control group of long-term studies and other studies where individuals were identified (Table 1). The 2 data sets were consistent; hence, we included all density data in our analysis. In order to expand coverage, density data were interpolated across the entire geographic range of the species (including regions in which hyenas were recently extirpated in central and southern Africa) using geographic midpoints of census sites and universal kriging in ArcGIS 10.6 (units = hyenas per km^2^; model = stable; nugget = 0.439; Sill = 0.2855; Major Range = 1.928; lag = 12; Cressie 1993; Esri 2018). Four density categories were identified using Jenks natural breaks (Jenks 1963). Exploratory models indicated a significant relationship between skull size and the lowest density category but did not show significant relationships between skull size and any of the other 3 density categories. Consequently, the number of density categories was reduced to 2: low (0.0045 to 0.02 Spotted Hyena/km^2^; *n* = 60); and high (0.03 to 1.65 Spotted Hyena/km^2^; *n* = 260), thereby decreasing the number of parameters in the model and simplifying interpretation.

**Table 1.** Locality, country, population density estimate (hyenas/km^2^), years(s) of study, study methods, and source for Spotted Hyena (*Crocuta crocuta*) densities used in this analysis (see Fig. 3 for map). NP = National Park; GR = Game Reserve. Long-term studies and studies where individuals were identified are denoted with an *.

Locality	Country	Density (km^2^)	Year(s)	Methods	References
Pendjari NP	Benin	0.02	2001 to 2009	Camera traps	Sogbohossou and Téhou (2007 )*
Chobe NP	Botswana	0.44	1986 to 1988	Direct observations, radio collars	Cooper (1989)*
Moremi GR	Botswana	0.14	2007 to 2010	Audio playback	Cozzi et al. (2013)
Benoue ecosystem	Cameroon	0.06	2015	Audio playback	Bauer et al. (2016)
Tigray (Wukro Dist.)	Ethiopia	0.52	2011	Audio playback, GPS collars	Yirga et al. (2013)
Tigray (Enderta Dist.)	Ethiopia	0.8	2012	Direct observations, road transects, calling stations	Yirga et al. (2017)
Mole NP	Ghana	0.12	2006 to 2009	Camera traps, patrol-based monitoring	Burton (2010)*
Masai Mara NR	Kenya	0.86	1979 to 1983	Direct observations, identification of individuals	Frank (1986)*
Aberdare NP	Kenya	1.34	1986 to 1987	Audio playback to bait stations	Sillero-Zubiri and Gottelli (1992)
Masai Mara	Kenya	0.94	1988 to 1992	Direct observations, identification of individuals	Watts and Holekamp (2008)*
Amboseli NP	Kenya	1.65	2003 to 2005	Direct observations, identification of individuals	Watts and Holekamp (2008)*
Namib-Naukluft NP	Namibia	0.005	1976 to 1977	Direct observations, identification of individuals	Tilson et al. (1980)*
Namib-Naukluft NP	Namibia	0.009	1977 to 1979	Direct observations, identification of individuals	Tilson and Henschel (1986)*
Etosha NP	Namibia	0.05	1986	Direct observations, counts at waterholes, radio collars	Gasaway et al. (1989)
Etosha NP	Namibia	0.02	2008	Audio playback	Trinkel (2009)
Odzala-Kotoua	Republic of Congo	0.46	1975 to 1977	Audio playback to bait stations	Whatley and Brooks (1978)*
Odzala-Kotoua NP	Republic of Congo	0.16	2007	Camera traps, track surveys	Henschel et al. (2014)*
Timbavati GR	South Africa	0.48	1973 to 1975	Observations of foraging; examination of scats and regurgitations	Bearder (1977)
Kruger NP	South Africa	0.32	1974 to 1975	Counts of culled individuals, aerial surveys	Smuts (1978)
Hluhluwe NP	South Africa	0.46	1975 to 1977	Audio playback to bait stations	Whatley and Brooks (1978)
Umfolozi GR	South Africa	0.36	1979 to 1981	Bait stations	Whateley (1981)
Kalahari Gemsbok NP	South Africa	0.009	1979 to 1984	Direct observations individually identified hyenas	Mills (1984)*
Kruger NP	South Africa	0.13	1982 to 1984	Camera traps, examination tracks, direct observations	Henschel and Skinner (1987)
Kruger NP	South Africa	0.19	1984 to 1989	Audio playback	Mills et al. (2001)
Mkuze GR	South Africa	0.13	1989	Radio collars	Skinner et al. (1992)*
Hluhluwe-iMfolozi	South Africa	0.36	2003 to 2004	Audio playback	Graf et al. (2009)
Serengeti	Tanzania	0.17	1965 to 1967	Direct observations, identification of individuals	Kruuk (1972)*
Ngorongoro	Tanzania	1.54	1965 to 1967	Direct observations, identification of individuals	Kruuk (1972)*
Selous GR	Tanzania	0.31	1991 to 1996	Audio playback	Creel and Creel (2002)
Ngorongoro	Tanzania	0.59	1996	Direct observations, identification of individuals	Höner et al. (2005)
Liuwa Plains NP	Zambia	0.33	1995 to 1999	Examination of scat	Purchase (2004)
Hwange NP	Zimbabwe	0.07	1999 to 2003	Radio collars, audio playback	Salnicki (2004)

### Statistical analysis

To assess sexual dimorphism, mean female-to-male skull centroid size ratio was first calculated for the entire sample (Ralls 1976). Because sexual size dimorphism was extremely small relative to size variation related to geography, sexes were pooled for subsequent geographic analyses.

An analysis of regional differences in sexual dimorphism was conducted to determine whether sexual size dimorphism varies across the range of the species, and whether sexual size dimorphism is greatest where population density—and thus presumably intrasexual competition—is also highest. The sample was divided into 3 geographic segments reflecting African Union designations (Oxfam and AfriMap 2010): eastern (east of the Nile and the East African lake system and dominated by tropical and subtropical grassland, savannas, and shrublands); western (west of the Nile and the East African lake system, and dominated by a mix of dense tropical forest and tropical and subtropical grassland, savannas, and shrublands), and southern (south of the East African lake system, and dominated by deserts and xeric shrubland). The single specimen from Mali was not included in this analysis because it was geographically distant from all others, with its nearest neighbor 2,714.4 km away (Breunig et al. 2000). The mean female-to-male skull centroid size ratio was calculated for each geographic region.

Geographic parameters (latitude and longitude) are difficult to model because ecological responses to spatial change are often nonlinear (Peters et al. 2007), such that a small change in the driving variable (geography) can have a large but discontinuous influence on the response variable. To accommodate this, we first quantified the spatial component of skull centroid size by determining the best-fit combination of spatial variables that contributed significantly to explaining variation in size, and then incorporated bioclimatic and density parameters into the analysis (Legendre and Legendre 1998; Botes et al. 2006; Cardini et al. 2007). The spatial component was modeled using a fourth-order polynomial, where *x* and *y* represent longitude and latitude, respectively. A global model of centroid size was regressed onto all higher-order polynomials for geography. We used the function pdredge from the R package “MuMIn” to perform an automated model selection with combinations (subsets) of fixed-effects terms from the global model to find the model with the most support using Akaike Information Criterion (AIC; Sakamoto et al. 1986). The model with the most support was used as the spatial component in subsequent analyses.

A model was built for each view of the skull by combining sex, temperature, precipitation, land cover, and density parameters with the view-specific spatial component to determine how each influences skull centroid size. Variance inflation factor analysis (vif package “car”) detected multicollinearity in all 3 views, so, following recommendations in Zuur et al. (2010), a backward stepwise variable selection was performed using AIC to address parameters problematic enough to impact the model. All impactful parameters were higher-order polynomials for geography. A reduced model for each view was constructed excluding the impactful parameters from the spatial component, and the reduced models were used in all subsequent analyses. We examined residuals and fitted values to determine whether assumptions of normality (Shapiro–Wilk), equal variance, and independence (Durbain Watson) were violated (Royston 1995; Fox and Wyrick 2008). We also examined the data using Cook’s distance (Fox and Wyrick 2008) and the function acf in the R package “forecast” (Venables and Ripely 2002) to identify any influential outliers or significant autocorrelations. All model analyses were performed in r version 3.4 (r Core Team 2017). To visualize size patterns across geography, centroid size was divided into 3 categories: small (315 to 360); medium (361 to 382); and large (383 to 422) using Jenks natural breaks and mapped using specimen locality data in ArcGIS Pro 2.9.4 (Esri 2020).

## Results

### Sexual dimorphism

Sex was a predictor of skull size, with females having significantly larger crania than males in ventral (*P*= 0.01; Table 2) and lateral views (*P* = 0.01; Table 3). There was no significant sex difference in lateral mandible size (*P* = 0.55; Table 4). Though statistically significant, actual size differences between female and male crania were very small; the mean female-to-male size ratio for our sample was 1.012 for ventral crania (females 115, males 122), 1.011 for lateral crania (females 114, males 122), and 1.007 for lateral mandibles (females 121, males 125; Table 5).

**Table 2.** Regression of *Crocuta crocuta* ventral cranium size onto geographic, environmental, and density variables: estimate, standard error (SE), *t*-value, and *P*-values. Residual SE = 13.61 on 295 degrees of freedom (df); multiple *R*-squared = 0.4476; adjusted *R*-squared = 0.4026; *F*-statistic = 9.958 on 24 and 295 df, *P* < 0.001 for full model. *X* represents longitude and *Y* represents latitude. For factor variables, sex female, density low, and vegetation type forest were set at reference levels. *P*-values less than 0.05 are denoted with an *.

Variables	Estimate	SE	*t*	*P*
(intercept)	−213.905	143.160	−1.494	0.136
*X*	65.994	15.527	4.250	0.000*
*X* ^2^	−2.216	0.528	−4.195	0.000*
*X* ^3^	0.024	0.006	4.095	0.000*
*Y*	−10.380	4.158	−2.497	0.013*
*Y* ^2^	0.843	0.737	1.144	0.254
*Y* ^3^	0.179	0.048	3.722	0.000*
*Y* ^4^	0.010	0.004	2.218	0.027*
*X × Y* ^2^	−0.066	0.050	−1.309	0.191
*X × Y* ^3^	−0.012	0.003	−3.804	0.000*
*X × Y* ^4^	−0.001	0.000	−2.070	0.039*
*X* ^2^ *× Y*	0.035	0.012	2.795	0.006*
*X* ^2^ *× Y* ^2^	0.001	0.001	1.412	0.159
*X* ^2^ *× Y* ^3^	0.000	0.000	3.607	0.000*
*X* ^2^ *× Y* ^4^	0.000	0.000	1.893	0.059*
*X* ^3^ *× Y*	−0.001	0.000	−2.893	0.004*
*X* ^3^ *× Y* ^4^	0.000	0.000	−1.723	0.086
Sex male	−4.766	1.829	−2.606	0.010*
Sex unknown	−6.006	2.195	−2.736	0.007*
Annual mean temperature	−0.028	0.032	−0.882	0.379
Annual precipitation	−0.009	0.005	−1.713	0.088
Density high	−19.787	4.888	−4.048	0.000*
Mixed forest and grassland	2.781	2.415	1.151	0.250
Grassland	1.448	3.277	0.442	0.659
Bare ground	7.859	7.182	1.094	0.275

**Table 3.** Regression of *Crocuta crocuta* lateral cranium size onto geography, environmental, and density variables: estimate, standard error (SE), *t*-value, and *P*-values. Residual SE = 39.75 on 299 degrees of freedom (df); multiple *R*-squared = 0.5758; adjusted *R*-squared = 0.5488; *F*-statistic = 21.36 on 19 and 299 df, *P* < 0.001 for full model. *X* represents longitude and *Y* represents latitude. For factor variables, sex female, density low, and vegetation type forest were set at reference levels. *P*-values less than 0.05 are denoted with an *.

Variables	Estimate	SE	*t*	*P*
(intercept)	837.393	30.003	27.910	0.000*
*Y*	−42.045	20.725	−2.029	0.043*
*Y* ^3^	0.230	0.112	2.042	0.042*
*X* ^2^ *× Y*	−0.232	0.087	−2.662	0.008*
*X* ^2^ *× Y* ^3^	0.001	0.001	2.241	0.026*
*X* ^2^ *× Y* ^4^	0.000	0.000	3.244	0.001*
*X* ^3^	−0.001	0.000	−1.868	0.063
*X* ^3^ *× Y*	0.003	0.001	2.528	0.012*
*X* ^3^ *× Y* ^3^	0.000	0.000	−2.016	0.045*
*X × Y*	6.044	2.361	2.560	0.011*
*X × Y* ^3^	−0.034	0.015	−2.301	0.022*
*X × Y* ^4^	0.000	0.000	−3.289	0.001*
Sex male	−12.994	5.278	−2.462	0.014*
Sex unknown	−9.635	6.269	−1.537	0.125
Annual mean temperature	−0.710	0.077	−9.255	0.000*
Annual precipitation	−0.051	0.011	−4.535	0.000*
Density high	−54.211	10.855	−4.994	0.000*
Mixed forest and grassland	−34.496	6.707	−5.143	0.000*
Grassland	−49.270	8.497	−5.798	0.000*
Bare ground	−68.930	19.063	−3.616	0.000*

**Table 4.** Regression of *Crocuta crocuta* lateral mandible size onto geography, environmental, and density variables: estimate, standard error (SE), *t*-value, and *P*-values. Residual SE = 19.15 on 307 degrees of freedom (df); multiple *R*-squared = 0.5254; adjusted *R*-squared = 0.4883; *F*-statistic = 14.16 on 24 and 307 df, *P* < 0.001 for full model. *X* represents longitude and *Y* represents latitude. For factor variables, sex female, density low, and vegetation type forest were set at reference levels. *P*-values less than 0.05 are denoted with an *.

Variables	Estimate	SE	*t*	*P*
(intercept)	304.102	85.667	3.550	0.000*
*Y*	27.247	10.142	2.686	0.008*
*Y* ^2^	1.630	0.497	3.283	0.001*
*Y* ^3^	−0.084	0.032	−2.667	0.008*
*Y* ^4^	−0.006	0.002	−3.912	0.000*
*X*	31.276	10.266	3.046	0.003*
*X* ^2^	−1.105	0.374	−2.954	0.003*
*X* ^2^ *× Y*	0.118	0.043	2.722	0.007*
*X* ^2^ *× Y* ^2^	0.003	0.001	3.273	0.001*
*X* ^2^ *× Y* ^3^	0.000	0.000	−2.874	0.004*
*X* ^2^ *× Y* ^4^	0.000	0.000	−3.911	0.000*
*X* ^3^	0.012	0.004	2.770	0.006*
*X* ^3^ *× Y*	−0.001	0.001	−2.740	0.006*
*X × Y*	−3.100	1.159	−2.674	0.008*
*X × Y* ^2^	−0.125	0.040	−3.150	0.002*
*X × Y* ^3^	0.006	0.002	2.671	0.008*
*X × Y* ^4^	0.001	0.000	3.773	0.000*
Sex male	−1.516	2.518	−0.602	0.548
Sex unknown	−2.283	3.005	−0.760	0.448
Annual mean temperature	0.063	0.042	1.487	0.138
Annual precipitation	0.001	0.007	0.116	0.908
Density high	−6.570	6.173	−1.064	0.288
Mixed forest and grassland	8.767	3.235	2.710	0.007*
Grassland	−0.249	3.992	−0.062	0.950
Bare ground	14.459	9.503	1.522	0.129

**Table 5.** *Crocuta crocuta* mean centroid size, standard deviation, and sample size for different regions of Africa for 3 skull views: ventral cranium (ventral); lateral cranium (lateral); and lateral mandible (mandible). Eastern includes east of the Nile and the East African lake system; western includes west of the Nile and the East African lake system; and southern includes south of the East African lake system. Mean female/mean male centroid size and sample sizes for females (*n*) and males (*n*).

	Skull view	Mean	SD	*n*	Mean female/male	Female *n*; male *n*
Total Africa	Ventral	368.89	±17.30	331	1.012	115; 122
	Lateral	539.77	±59.25	337	1.011	114; 122
	Mandible	575.65	±26.77	332	1.007	121; 125
Western	Ventral	385.97	±16.01	50	1.029	10; 12
	Lateral	541.16	±24.55	52	1.005	11; 12
	Mandible	596.05	±25.79	58	0.997	13 12
Eastern	Ventral	363.39	±13.38	256	1.015	100; 102
	Lateral	539.87	±65.89	262	1.012	96; 103
	Mandible	566.61	±19.38	248	1.010	102; 104
Southern	Ventral	390.04	±15.58	24	0.960	4; 8
	Lateral	536.07	±26.76	22	1.020	4; 8
	Mandible	616.81	±28.51	24	1.025	4; 9

Analysis of regional differences did not indicate geographic variation in sexual size dimorphism and provided no evidence that degree of sexual dimorphism is positively correlated with hyena density (Table 5). Females were slightly larger than males for all skull views in the eastern population and for 2 of 3 skull views in western and southern populations. Males were slightly larger than females for 1 view (mandible) in western populations and 1 (ventral) in southern populations (Table 5). However, sample sizes for western and southern populations were much smaller than for the eastern geographic region.

### Geography

We observed a considerable range in adult skull size across geography, with the smallest skull being 25% smaller than the largest skull. Geographic parameters (i.e., latitude and longitude) were strongly predictive in explaining variation in skull centroid size in our models with 68.75% of geographic parameters significant in ventral view (*P* < 0.05), 90.91% in lateral view (*P* < 0.05), and 100% in lateral view of mandible (*P* < 0.05; Tables 2–4). Individuals with small skulls were clustered between −5.00° and 10.00° latitude and east of 28.50° longitude (Fig. 1). The eastern boundary of this cluster corresponds with the Albertine Rift, which is the western branch of the East African rift system (Ebinger 1989). Specimens from near Aberdare National Park in central Kenya, east of the East African Rift Valley, had the smallest mean ventral crania, lateral crania, and lateral mandibles, while the largest skull was from Botswana in southern Africa.

### Bioclimatic variables

Annual mean temperature and annual precipitation were significant predictors of lateral cranium size in Spotted Hyenas, with smaller centroid sizes occurring in drier (*P* < 0.001) and cooler (*P* < 0.001) regions (Table 3). These variables were not, however, significant predictors of centroid size of either the ventral cranium (Table 2) or lateral mandible (Table 4).

Vegetation type was a significant predictor of lateral cranium (Table 3) and lateral mandible (Table 4) centroid size, but not of ventral cranium centroid size (Table 2). Lateral crania (*P* < 0.001) were smaller in more closed habitats with trees (i.e., forest or mixed forest and grassland) than in more open habitats (i.e., grassland and bare ground), whereas lateral mandibles were largest in mixed forests and grassland. This contrasting relationship between size and habitat variables in lateral crania versus lateral mandibles seems surprising because these structures are expected to coevolve, as they function together as a feeding apparatus. However, centroid size is not a linear measurement; rather, it captures distances of each landmark from the centroid of the landmark configuration. Thus, changes in mandible shape, such as in the curvature of the coronoid process or the arch of the interparietal, can affect the magnitude of this metric. Additionally, the mandible of mammals is very plastic and has been shown to change shape with changes in dietary hardness (Meyer 1987; Mavropoulos et al. 2004; Renaud et al. 2010; Scott et al. 2014). Spotted hyenas are highly durophagous, relying on bone-cracking to access marrow when prey are scarce, and may experience plastic shape change in the mandible over the course of their lifetime.

### Hyena density

Population density was a significant predictor of ventral (*P* < 0.001) and lateral (*P* < 0.001) cranium centroid size; larger crania came from regions where population density is lower (Tables 2 and 3). Population density was not, however, a significant predictor of lateral mandible centroid size (*P* = 0.288; Table 4).

## Discussion

Studying patterns of size variation across geographic regions can provide insight into the interplay of environmental and social factors that drive morphological evolution. This study examines geographic variation in the size of Spotted Hyena skulls. Our aim was to identify how size co-varies with sex, bioclimatic variables, and population density in nature.

### Sexual dimorphism

Our finding that female spotted hyenas have larger crania than males is in agreement with several previous studies (Matthews 1939; Kruuk 1972; Swanson et al. 2013). The ratio of mean female to mean male size was slightly, but significantly, greater than 1.00 for ventral and lateral crania (i.e., 1.012 and 1.011, respectively). These findings are comparable to those of Ralls (1976), who reported a 1.04 female-to-male ratio for mean head and body length in this species. They support the hypothesis proposed by Rensch (Rensch’s Rule), which predicts that in species with female-biased sexual size dimorphism, the size difference between sexes will be small (Rensch 1950; Bidau and Martinez 2016). Our results also suggest that Swanson et al. (2013) were correct in concluding that the size difference between male and female spotted hyenas is very small and thus not consistently detected when sample sizes are small.

Historically, female-biased sexual size dimorphism has not been consistently detected in Spotted Hyenas, with some authors finding no sexual size dimorphism or male-biased sexual size dimorphism (Buckland-Wright 1969; Skinner 1976; Sillero-Zubiri and Gottelli 1992; Arsznov et al 2010; Sakai et al. 2011; Mann et al 2018). One plausible explanation for these different results is that there is regional variation in sexual size dimorphism. Although we did not test this hypothesis directly (e.g., by examining additional specimens from the same localities as previous studies), our broad regional analysis suggests that sexual dimorphism in skull size varies little, if at all, with geography. Thus, the likely explanation for inconsistent findings is small sample size—only one of these previous studies had more than 30 Spotted Hyenas of each sex (35 females, 44 males; Mann et al. 2018).

### Geography and bioclimatic factors

We found that the smallest individuals occur in equatorial East Africa. The smallest, on average, are within 160 km of the Aberdare National Park. Due to high elevation, these Spotted Hyenas experience cold temperatures despite living near the equator. If patterns in size were driven by thermoregulation, as proposed by Bergmann (1847), we would expect Spotted Hyenas in the Aberdare National Park to be larger. Our data are not consistent with Bergmann’s Rule in that individuals are larger everywhere else in Africa, including in low-elevation equatorial regions west of the Albertine Rift. The smallest Spotted Hyena skulls are found between −5.00° and 10.00° latitude and east of 28.50° longitude, with larger individuals found elsewhere (Fig. 1).

Previous researchers have described geographic clines in body size of Spotted Hyenas. Kruuk (1972) reported a northwest-to-southeast cline with larger Spotted Hyena skulls found in Queen Elizabeth Park in Uganda than in the Serengeti. Sillero-Zubiri and Gottelli (1992) described a south-to-east cline in body mass with the lightest Spotted Hyenas being found in the Aberdare Forest. Kurten (1957) reported a 2-direction cline, extending north and south from the equator for a sample of mixed extant African *C. crocuta* and late Pleistocene Syrian and European populations of *C. spelaea*, which he considered to be a single species. Carnassial lengths were smallest in the equatorial belt and increased gradually to the north and south (Kurten 1957). Klein and Scott (1989) described a tendency for Spotted Hyena carnassial length to increase with latitude in present-day Africa, suggesting that Spotted Hyena body size is inversely related to temperature, as predicted by Bergmann’s Rule. It is possible that the Kurten (1957) and Klein and Scott (1989) plots of lower carnassial lengths against latitude for Spotted Hyenas were overly influenced by the large numbers of individuals from east Africa. However, we cannot directly compare our results to theirs, as they did not report longitude for specimens. We would caution against using *C. crocuta* carnassial length as an independent gauge of Pleistocene temperature variation, as our large sample of modern *C. crocuta* did not conform well to Bergmann’s Rule.

In contrast to the predictions of Bergmann’s Rule, we found that annual mean temperature and annual precipitation were significant predictors of lateral cranium size, with smaller skulls found in cooler and dryer areas (Table 3). We expected larger skulls and, by proxy, larger individuals in drier areas because larger bodies retain moisture better than smaller bodies, which have a higher surface area-to-volume ratio (Hudson and White 1985; Hill et al. 2004). Perhaps finding smaller individuals in drier areas reflects the greater importance of dissipating heat over retaining moisture (Hudson and White 1985; Hill et al. 2004). Alternatively, the pattern of smaller lateral crania in dry and cool areas may be an artifact of some other relationship related to geographic area that we did not measure. Other factors including seasonality of precipitation, temperature fluctuations, or prey abundance could potentially have a greater impact on Spotted Hyena size than our bioclimatic variables. We had incomplete sampling in western tropical Africa and northern Ethiopia due to sparse representation in museum collections. These collections mainly comprised opportunistic and donated specimens, potentially introducing size biases. Future studies should expand the sample size specifically from these regions.

We expected land cover to influence Spotted Hyena size because land cover provides habitat for prey, influences prey abundance, and affects predator navigation and perception. We found smaller lateral crania in forest, and mixed grassland and forest, than in grassland or bare ground. However, we did not find a significant relationship between ventral cranium size and land cover. The sagittal and nuchal crests, captured by landmarks on the lateral view of the skull, provide surface area for the attachment of the temporalis muscle, and the temporalis muscle functions in jaw closure. Larger sagittal and nuchal crests allow for larger muscle attachment, generating a more powerful bite. Spotted Hyenas in grassland or bare ground habitats may need stronger bite forces to subdue prey. The ventral cranium landmarks capture zygomatic arch variation influencing masseter muscle attachment. As the masseter muscle functions in lateral jaw movement, it is less vital for carnivoran feeding; the ventral skull view may have minimal correlation with the prey encountered in different vegetation types.

Sillero-Zubiri and Gottelli (1992) also found small spotted hyenas in the forest of the Aberdares. Open grassland habitats in East Africa have higher densities of prey than the montane forest of the Aberdares (Sinclair and Arcese 1995; Massey et al. 2014), yet Spotted Hyena density in the Aberdares is very high, exceeding 1 animal per km^2^ (Sillero-Zubiri and Gottelli 1992). Fewer prey and higher densities of spotted hyenas result in less food per capita. Thus, smaller Spotted Hyenas in forests may be a consequence of limited resources in forested habitats, and combined with higher densities of Spotted Hyenas provide empirical evidence for the energetic equivalence rule (Damuth 1981).

### Hyena density

Skull size of Spotted Hyenas varies with geography but does not adhere to strict geographical and ecological rules such as Bergmann’s Rule. We found that Spotted Hyena crania were smaller in areas characterized by higher population densities (Tables 2 and 3). This pattern is congruent with the energetic equivalence rule (Damuth 1981) and is consistent with what has been seen in some other mammals (Red Fox, Cavallini 1995; Brown Bear, Zedrosser et al. 2006; primates, Clutton-Brock and Harvey 1977; Reindeer, Skogland 1983; Moose, Sand et al. 1995; White-tailed Deer, Simard et al. 2008), suggesting that intraspecific competition for resources is an important driver of cranium size in Spotted Hyena. The smallest skulls for all 3 views were from East Africa where clan sizes are known to exceed 100 individuals (Green et al. 2018).

Not only do spotted hyenas compete with conspecifics for resources, but with potentially 22 other carnivore species as well (Caro and Stoner 2003). Kleptoparasitism of Spotted Hyena kills by lions is common throughout Africa, and Spotted Hyenas living at lower lion densities have greater lifetime reproductive success than those living at higher lion densities, likely reflecting higher rates of food intake (Höner et al. 2002; Watts and Holekamp 2008). Thus, access to resources for Spotted Hyena is further diminished due to intraguild competition. We suggest that future studies examine the impact of intraguild competition on Spotted Hyena size in relation to the energetic equivalence rule.

In summary, our study reveals sexual size dimorphism in Spotted Hyenas, with females having slightly larger skulls. This size difference, although small, is statistically significant. We found no compelling evidence for geographic variation in sexual dimorphism. The smallest Spotted Hyenas inhabit an East African region near the lower Rift Valley, while larger individuals are found elsewhere. High population density is positively correlated with small skull size, possibly reflecting increased resource competition. These findings indicate that geographic variation in Spotted Hyena skull size aligns more closely with the energetic equivalence rule than Bergmann’s Rule.

## Supplementary data

Supplementary data are available at *Journal of Mammalogy* online.

**Supplementary Data SD1.**—Ventral landmarks definitions.

**Supplementary Data SD2.**—Lateral landmarks definitions.

**Supplementary Data SD3.**—Mandible landmarks definitions.

**Supplementary Data SD4.**—*Crocuta crocuta* specimens ventral cranium.

**Supplementary Data SD5.**—*Crocuta crocuta* specimens lateral cranium.

**Supplementary Data SD6.**—*Crocuta crocuta* specimens mandible.

gyae023_suppl_Supplementary_Datas_SD1

gyae023_suppl_Supplementary_Datas_SD2

gyae023_suppl_Supplementary_Datas_SD3

gyae023_suppl_Supplementary_Datas_SD4

gyae023_suppl_Supplementary_Datas_SD5

gyae023_suppl_Supplementary_Datas_SD6
