# Supplementary material for: Spotted Hyena skull size variation across geography favors the energetic equivalence rule over Bergmann’s Rule
Source: J Mammal. 2024 Apr 24;105(4):910–23. doi: 10.1093/jmammal/gyae023 (PMC11285150; doi:10.1093/jmammal/gyae023)
Supplement: gyae023_suppl_Supplementary_Datas_SD1 [file gyae023_suppl_supplementary_datas_sd1.docx]

**Supplementary Data SD1**.—Ventral landmarks definitions.

| Landmark | Definition |
| --- | --- |
| 1 | Juncture between the incisors on the premaxilla |
| 2 | Premaxilla-maxilla suture intersection with the medial edge of the left canine |
| 3 | Most posterior point of the left incisive foramen |
| 4 | Most posterior point of the right incisive foramen |
| 5 | Premaxilla-maxilla suture intersection with the medial edge of the right canine |
| 6 | Posterior edge of premaxilla-maxilla suture on the palate |
| 7 | Center of Maxilla-palatine midline suture |
| 8 | Center of left fourth premolar |
| 9 | Center of right fourth premolar |
| 10 | Posterior edge of the midline suture between the left and right palatine. |
| 11 | Most posterior edge of the left maxilla-jugal suture |
| 12 | Most posterior edge of the right maxilla-jugal suture |
| 13 | Most posterior edge of the left jugal-squamosal suture |
| 14 | Most posterior edge of the right jugal-squamosal suture |
| 15 | Most anterior point of the foramen magnum |
| 16 | Posterior end of the left maxilla-palatine suture |
| 17 | Posterior end of the right maxilla-palatine suture |
| 18 | Center of left, jugular canal |
| 19 | Center of right, jugular canal |
| 20 | Most anterior point of left retroarticular process |
| 21 | Most anterior point of right retroarticular process |
| 22 | Posterior edge of left second premolar |
| 23 | Posterior edge of right second premolar |
| 24 | Most distal point of the left external auditory meatus |
| 25 | Most distal point of the right external auditory meatus |
