# Supplementary material for: Spotted Hyena skull size variation across geography favors the energetic equivalence rule over Bergmann’s Rule
Source: J Mammal. 2024 Apr 24;105(4):910–23. doi: 10.1093/jmammal/gyae023 (PMC11285150; doi:10.1093/jmammal/gyae023)
Supplement: gyae023_suppl_Supplementary_Datas_SD2 [file gyae023_suppl_supplementary_datas_sd2.docx]

**Supplementary Data SD2**.—Lateral landmarks definations.

| Landmark | Definition |
| --- | --- |
| 1 | Anterior edge of the third incisor |
| 2 | Anterior edge of canine |
| 3 | Posterior edge of canine |
| 4 | The most posterior part of the infraorbital foramen |
| 5 | The intersection of the maxilla, lacrimal and jugal |
| 6 | The most lateral projection of post-orbital process |
| 7 | Most dorsal anterior part of the squamosal |
| 8 | Most ventral posterior part of the jugal |
| 9 | The most ventral-posterior point of the jugal-maxilla suture |
| 10 | The most posterior edge of the suture of palatine and pterygoid process |
| 11 | Suture of the squamosal and occipital inside the auditory meatus |
| 12 | Anterior upper edge of the occipital condyle |
| 13 | Posterior most edge of the sagittal crest |
| 14 | Anterior edge of the nasal-premaxilla suture |

32 Semilandmarks along curve of dorsal cranium, 14 to 13
