# Supplementary material for: Spotted Hyena skull size variation across geography favors the energetic equivalence rule over Bergmann’s Rule
Source: J Mammal. 2024 Apr 24;105(4):910–23. doi: 10.1093/jmammal/gyae023 (PMC11285150; doi:10.1093/jmammal/gyae023)
Supplement: gyae023_suppl_Supplementary_Datas_SD3 [file gyae023_suppl_supplementary_datas_sd3.docx]

**Supplementary Data SD3.**—Mandible landmarks definitions.

| Landmark | Definition |
| --- | --- |
| 1 | Anterior edge of third incisor |
| 2 | Anterior edge of canine |
| 3 | Posterior edge of canine |
| 4 | Dorsal apex of the coronoid process |
| 5 | Most posterior projection of the coronoid process |
| 6 | Anterior edge of the mandibular condyle, distal to the vertical plane of the coronoid |
| 7 | Posterior most edge of the mandibular condyle |
| 8 | Posterior most point of the articular process |
| 9 | Intersection of the mandibular body and ramus |
| 10 | Intersection of anterior margin of first incisor with the dentary |
|  |  |

32 Semilandmarks along ventral curve of the mandible, 10 to 8

11 Semilandmarks along posterior curve between articular process and mandibular condyle, 8 to 7

16 Semilandmarks along posterior curve between mandibular condyle and coronoid process, 6 to 5

17 Semilandmarks along anterior curve of ramus, 4 to 9
