# Supplementary material for: Spotted Hyena skull size variation across geography favors the energetic equivalence rule over Bergmann’s Rule
Source: J Mammal. 2024 Apr 24;105(4):910–23. doi: 10.1093/jmammal/gyae023 (PMC11285150; doi:10.1093/jmammal/gyae023)
Supplement: gyae023_suppl_Supplementary_Datas_SD4 [file gyae023_suppl_supplementary_datas_sd4.docx]

**Supplementary Data SD4.**—*Crocuta crocuta* specimens ventral cranium.

AMNH: 114227, 114256, 165118, 165119, 187769, 187771, 187772, 187776, 187777, 187779, 187782, 20809, 20810, 216355, 27765, 27767, 52059, 52060, 52063, 52064, 52065, 52068, 52069, 52097, 54243, 54244, 55467, 81833, 83591, 83592, 83593; BM: 3441136, 314113, 39383, 1031, 31822, 862, 18927, 19613, 2854, 21291030, 233414, 233415, 233416, 233419, 25124233, 27299, 27738A, 27738, 281163, 291138, 3012182, 311211, 3441137, 3441138, 3441139, 3441140, 38101847, 385102, 385103, 39339, 39340, 39342, 39343, 39344, 39345, 39346, 39348, 39349, 39351, 39353, 39355, 39356, 39358, 39360, 39361, 39362, 39363, 39364, 39366, 39368, 39369, 39370, 39373, 39375, 39376, 39378, 39381, 39382, 39387, 39388, 39389, 39390, 39391, 39394, 39395, 39396, 39397, 39399, 39400, 39401, 39402, 39403, 39404, 39407, 39408, 39409, 39410, 39411, 39412, 39413, 39414, 39416, 39417, 39419, 39420, 39421, 39422, 39423, 39424, 39425, 39427, 39428, 39429, 39430, 39431, 39432, 39433, 39435, 39437, 58208, 62706, 62707, 96114, 9 6 1 14, 92 8 1 4; Cambridge: K4062, K4065, K4067; CM: 20871, 5862, 63108; FMNH: 104021, 104981, 127825, 127826, 127829, 135072, 32933, 34582, 34583, 73034, 73035, 98739, 98952; MSU2: 37464-BFT, 37465-ECO, 37466-VGS, 36550, 36551, 36552, 36558, 36567, 36568, 36569, 36570, 36571, 36581, 12391, 22401, 26055, 2714, 35852, 35853, 35854, 35856, 35857, 35858, 36008, 36011, 36074, 36077, 36078, 36079, 36080, 36083, 36084, 36094, 36160, 36161, 36163, 36165, 36168, 8048; MVZ: 165159, 165160, 165162, 165163, 165165, 165166, 165167, 165169, 165170, 165175, 165176, 165179, 165180, 165181, 165182, 173733, 173734, 173737, 173743, 173746, 173747, 173748, 173751, 173758, 173759, 173768, 173770, 173771, NMK: 175801, 184088, 184089, 2705, 3580, 7189, 7755, 7757, 7761, 7762, 7850; USNM: 164506, 164549, 20874, 163099, 163100, 163101, 163102, 163103, 164502, 181516, 181518, 181519, 181520, 181521, 181524, 181525, 181526, 181527, 181530, 181533, 181534, 182032, 182085, 182091, 182095, 182103, 182113, 182210, 201010, 239161, 367384, 367385, 368502, 429176; OSU: 11969, 11970, 4640, 4650, 4651, 4682, 5711, 5761; PMNHN-OM MNHNCA: 1896-450, 1894-54; PMNHN-OM MNHNZ: 1962-1537, 1972-400, 1996-2514, 1997-415; RBINS: 10250, 10336, 11799, 11801, 21278, 21302, 21436, 4612, 8632, 8633, 8634, 9480; RCSOM: 16.5, 137.41, 137.42, 137.43; RMCA: 11376, 11602, 11701, 12096, 12442, 14367, 14369, 14813, 16719, 17619, 18495, 18627, 1897, 2162, 22802, 2907, 36328, 36543, 36545, 3728, 3788, 3870, 5934, and 9292
