# Supplementary material for: Spotted Hyena skull size variation across geography favors the energetic equivalence rule over Bergmann’s Rule
Source: J Mammal. 2024 Apr 24;105(4):910–23. doi: 10.1093/jmammal/gyae023 (PMC11285150; doi:10.1093/jmammal/gyae023)
Supplement: gyae023_suppl_Supplementary_Datas_SD6 [file gyae023_suppl_supplementary_datas_sd6.docx]

**Supplementary Data SD6.**—*Crocuta crocuta* specimens mandible.

AMNH: 114226, 114227, 114256, 165118, 165119, 187769, 187771, 187772, 187776, 187777, 187782, 20809, 20810, 216355, 27765, 27767, 52059, 52060, 52063, 52064, 52065, 52068, 52069, 52097, 54243, 54244, 55467, 83591, 83592, 83593; BM: 34 4 1 134, 0 10 3 1, 0 3 18 22, 1 8 9 27, 15 3 6 90, 19 6 1 3, 2 8 5 4, 21 29 10 30, 23 3 4 15, 23 3 4 11, 23 3 4 14, 23 3 4 19, 24 8 3 4, 24 8 3 74, 25 12 4 233, 27 2 9 9, 27 7 3 8A, 27 7 3 8, 28 11 6 3, 29 11 3 8, 30 12 182, 31 1 2 11, 31 4 1 13, 34 4 1 136, 34 4 1 137, 34 4 1 138, 34 4 1 139, 34 4 1 140, 38 10 18 47, 38 5 10 2, 38 5 10 3, 39 337, 39 339, 39 340, 39 342, 39 343, 39 344, 39 345, 39 346, 39 348, 39 349, 39 351, 39 353, 39 356, 39 358, 39 359, 39 360, 39 361, 39 362, 39 368, 39 363, 39 364, 39 366, 39 369, 39 370, 39 373, 39 375, 39 376, 39 378, 39 381, 39 383, 39 385, 39 386, 39 387, 39 388, 39 389, 39 390, 39 391, 39 394, 39 395, 39 396, 39 397, 39 399, 39 400, 39 401, 39 402, 39 403, 39 404, 39 407, 39 408, 39 409, 39 410, 39 411, 39 412, 39 413, 39 414, 39 416, 39 417, 39 419, 39 420, 39 425, 39 421, 39 422, 39 423, 39 424, 39 427, 39 428, 39 429, 39 430, 39 431, 39 432, 39 433, 39 435, 39 437, 58 208, 59 272, 62 706, 62 707, 66 792, 9 6 1 14, 92 8 1 4; Cambridge: K4062, K4065, K4067; CM: 20871, 5862, 63108, 6827; FMNH: 104021, 104981, 127825, 127826, 127829, 32933, 34582, 34583, 73034, 73035, 93866, 98739, 98952; MSU: 36011, 225 VGS, 35852, 35854, 35856, 36008, 36077, 36079, 36083, 36084, 36163, 36165, 36168, 36550, 36551, 36552, 36558, 36567, 36568, 36569, 36570, 36571, 36581, 486 ECO, 897 BFT, 12391, 22401, 24292, 26055, 2714, 35853, 35857, 35858, 36074, 36078, 36080, 36094, 36160, 36161, 8048; NHM115, MVZ: 165170, 165160, 165162, 165163, 165165, 165167, 165169, 165175, 165176, 165179, 165180, 165181, 165182, 173733, 173734, 173737, 173741, 173743, 173745, 173746, 173751, 173768, 173770, 173771, 175801, 184088, 184089; NMK: 2703, 2705, 7189, 7755, 7757, 7761, 7762, 7850; USNM: 20874, 122544, 163099, 163100, 163101, 163102, 181520, 163103, 164502, 164506, 164549, 181516, 181518, 181519, 181521, 181524, 181525, 181526, 181527, 181530, 181533, 181534, 182032, 182085, 182091, 182095, 182103, 182105, 182113, 182117, 182210, 201010, 239161, 367384, 367385, 368502, 429176; PMNHN-AC MNHNCA1894-54; PMNHN-OM MNHNZ:1962-1537, 1972-400, 1996-2514; RBINS: 10250, 10336, 11799, 11801, 11804, 21278, 9480, 21302, 21436, 4612, 7705, 8632, 8633, 8634, 9967; RCSOM: 137.41, 137.42, 137.43, 16.5; RMCA: 11376, 11602, 11701, 12096, 12442, 14367, 14369, 16719, 17619, 17740, 18000, 18495, 18627, 1897, 19272, 19273, 2162, 22802, 2907, 36328, 36543, 3728, 3788, 384, 3870, 5934, 9292, 9579; OSU: 4650, 11969, 4640, 4651, 4682, 5711, and 5761
